# Optimisation of Bee Pollen Extraction to Maximise Extractable Antioxidant Constituents

**DOI:** 10.3390/antiox10071113

**Published:** 2021-07-12

**Authors:** Ivan Lozada Lawag, Okhee Yoo, Lee Yong Lim, Katherine Hammer, Cornelia Locher

**Affiliations:** 1 Cooperative Research Centre for Honey Bee Products Limited (CRC HBP), University of Western Australia, Agriculture North M085, Perth, WA 6009, Australia; ivan.lawag@research.uwa.edu.au (I.L.L.); katherine.hammer@uwa.edu.au (K.H.); 2 Division of Pharmacy, School of Allied Health, University of Western Australia, Curnow Building M315, Perth, WA 6009, Australia; okhee.yoo@research.uwa.edu.au (O.Y.); lee.lim@uwa.edu.au (L.Y.L.); 3 M Block QEII Medical Centre, School of Biomedical Sciences, University of Western Australia, Monash Ave, Perth, WA 6009, Australia

**Keywords:** bee pollen, Jarrah, Marri, extraction, 2-diphenyl-1-picrylhydrazyl (DPPH), ferric reducing antioxidant power (FRAP), total phenolic content (TPC), optimisation, antioxidant activity, review, multilevel factor analysis (MFA)

## Abstract

This paper presents the findings of a comprehensive review on common bee pollen processing methods which can impact extraction efficiency and lead to differences in measured total phenolic content (TPC) and radical scavenging activity based on 2,2-diphenyl-1-picrylhydrazyl (DPPH) and ferric reducing antioxidant power (FRAP) data. This hampers the comparative analysis of bee pollen from different floral sources and geographical locations. Based on the review, an in-depth investigation was carried out to identify the most efficient process to maximise the extraction of components for measurement of TPC, DPPH and FRAP antioxidant activity for two bee pollen samples from western Australia (Jarrah and Marri pollen). Optimisation by Design of Experiment with Multilevel Factorial Analysis (Categorical) modelling was performed. The independent variables included pollen pulverisation, the extraction solvent (70% aqueous ethanol, ethanol, methanol and water) and the extraction process (agitation, maceration, reflux and sonication). The data demonstrate that non-pulverised bee pollen extracted with 70% aqueous ethanol using the agitation extraction method constitute the optimal conditions to maximise the extraction of phenolics and antioxidant principles in these bee pollen samples.

## 1. Introduction

Flower pollen are the reproductive cells found in the stamen of plants which are transferred to the stigma of another plant via pollinating agents, such as bees, other insects, wind and water [1]. Bee pollen, on the other hand, is made by worker honeybees combining flower pollen, nectar and bee salivary constituents, and it is transferred to beehives in the form of pollen baskets attached to the bees’ hind legs [1,2]. Inside the hives, bee pollen is packed into honeycomb cells and covered with a layer of honey and wax to initiate fermentation to generate bee bread, which is the principal source of nutrients for honeybees [2]. Bee pollen, which is also known as apicultural, bee-collected or corbicular pollen, can be harvested for human consumption with the help of pollen traps. These traps are fixed at the entrance of beehives and collect pollen by stripping the pollen baskets from the hind legs of bees on entry to the hives [3].

Bee pollen provides bees with carbohydrates and other necessary nutrients such as proteins, fats, minerals and vitamins [4]. The secondary metabolite profiles of bee pollens vary significantly, reflecting the botanical and geographical origin as well as the climatic conditions, soil type and beekeeper activities [5,6]. The chemical composition of bee pollens typically includes 13–55% carbohydrates, 10–40% proteins and 1–13% lipids, alongside minor components such as minerals and trace elements, vitamins, carotenoids, phenolic compounds, flavonoids, sterols and terpenes [6,7,8]. Some even consider bee pollen to be the only complete food, as it contains all the essential amino acids needed by humans [6]. Bee pollen has been recommended as a natural nutraceutical due to its antimicrobial, antioxidant, anti-inflammatory, antiallergen, anticarcinogenic, antiradiation, antiulcer, hepatoprotective and chemopreventive properties. More recently, bee pollen has been found to modulate gut microbiota to promote gut health [6,7,8].

The antioxidant activity of bee pollen is mainly associated with the presence of polyphenols in the form of phenolic acids and flavonoids, which exert their antioxidant activity by neutralising free radicals through the donation of electron or hydrogen atoms [9]. This bioactivity has attracted considerable interest in recent years because of its association with anti-inflammatory, anti-cancer and also anti-aging effects [5]. Commonly, the determination of the antioxidant activity of honey and other bee products such as bee pollen involves the use of a range of popular colorimetric assays. These include the measurement of total phenolic content (TPC) [10,11,12,13,14], total flavonoid content (TFC) [13,14,15], free radical scavenging activity using the 2,2-diphenyl-1-picrylhydrazyl (DPPH) assay [4,12,13,14,15,16] or measuring the ferric reducing antioxidant power (FRAP) [4,10,17]. DPPH and FRAP assays, in particular, are widely used to determine the antioxidant activity of plant extracts and food products. These assays use stable redox reagents which are inexpensive and easy to prepare, they are quick and easy to perform, and the results are accurate, highly reproducible, and readily validated [18]. 

The determination of the antioxidant activity of bee pollen necessitates an extraction step guided by the premise of a high yield and minimal changes to the functional properties of the extract. It is, therefore, necessary to select an appropriate extraction method and solvent, based on sample matrix properties, the chemical properties of the analytes as well as potential matrix–analyte interactions [19]. Further parameters that require consideration are the number of extractions to be performed, the extraction time, the ratio of solvent to raw material, and the extraction pressure and temperature. As the polarity of bioactive polyphenols ranges from very polar to relatively non-polar, the extraction solvent plays a significant role in extraction efficiency [20]. Furthermore, pre-extraction processes such as pulverisation can also affect the extraction efficiency of bioactive principles, as powdered samples have smaller particle sizes and narrower particle size distribution, leading to improved surface contact with extraction solvents [21]. 

In order to determine an optimal extraction process for bee pollen that yields the maximum extraction of antioxidant constituents, a survey of the literature was carried out. A review of 101 papers published between 2003 and 2021 reporting the antioxidant activity of bee pollen found a wide range of extraction conditions (Table 1). Some, but not all, subjected the samples to pulverisation (31.7%). Extraction solvents also varied, though they were mainly of high polarity, with methanol (21.8%), 70% aqueous ethanol (13.0%), ethanol (10.1%) and water (10.1%) being the more commonly used solvents. As for the extraction process itself, maceration was found to be most widely used (34.8%), followed by sonication (26.1%), agitation (23.1%) and reflux extraction (1.5%).

It can be assumed that the adopted extraction process for a bee pollen sample, including the sample pre-treatment, extraction method and solvent, impacts the extraction efficiency of antioxidant constituents and consequently on the level of antioxidant activity measured. The objective of this study was to use the Design of Experiment approach to optimise an extraction process for bee pollen with a view to maximising the extraction of antioxidant constituents as measured by the DPPH, FRAP and TPC assays. The independent variables were sample pulverisation, extraction process (agitation, maceration, reflux or sonication) and extraction solvent (methanol, 70% aqueous ethanol, ethanol or water). Two different bee pollen samples from western Australia, Jarrah (*Eucalyptus marginata*) and Marri (*Corymbia calophylla*) bee pollen, were used as model bee pollen samples. 

## 2. Materials and Methods

### 2.1. Chemicals, Reagents and Pollen Samples

The chemicals and reagents were sourced as follows: Folin and Ciocalteu’s phenol reagent 2N, (F9252-1L, Lot No. SHBH4781V), 2,4,6-tris(2-pyridyl)-1,3,5-triazine (TPTZ ≥ 98.0%, Lot No. BCBW0518, CAS No. 3682-35-7), iron (III) chloride hexahydrate (FeCl_3_·6H_2_O ACS Reagent 97.0%, Lot No. BCBZ5998, CAS No. 10025-77-1), iron (II) sulphate heptahydrate (FeSO_4_·7H_2_O ACS reagent, ≥99.0%, Lot No. MKCJ9113, CAS No. 7782-63-0), fructose (C_6_H_12_O_6_ ≥ 99.0%, Lot No. SLBZ1343, CAS No. 57-48-7), and maltose (C_12_H_22_O_11_·H_2_O BioXtra, ≥99%, Lot No. SLCC4130, CAS No. 6363-53-7) from Sigma Aldrich Truganina, Victoria 3029 Australia.; sodium anhydrous carbonate (Na_2_CO_3_, LR, B.N. 334280, CA No. 497-19-8), glucose (D-glucose anhydrous, C_6_H_12_O_6_ 99.0%, Batch No. 314615, CAS No. 50-99-7) sucrose (C_12_H_22_O_11_ 99.0%, Batch No. 324472, CAS No. 57-50-1), ethanol (CH_3_CH_2_OH, B.N. 360221, 64-17-5), from Chem Supply, Port Adelaide, SA 5015, Australia; anhydrous sodium acetate (C_2_H_3_NaO_2_ ≥ 99.0%, Batch No. AF512004, CAS No. 127-09-3) and glacial acetic acid ≥ 99.0%, Batch No. AH602167, CAS No.64-19-7) were purchased from Ajax Finechem, Wollongong NSW 2500, Australia; hydrochloric acid (HCl, Pt. 3350-1813, CAS No. 7647-01-0) was obtained from Asia Pacific Specialty Chemicals Limited, Seven Hills NSW 2147, Australia; 2,2-diphenyl-1-picrylhydrazyl (C_18_H_12_N_5_O_6_, CAS No. 1898-66-4 was purchased from Fluka AG, Buchs, Sankt Gallen, Switzerland; trihydroxybenzoic acid (C_7_H_6_O_5_, Lot. No. 1278, CAS No. 149-91-7) from Ajax Chemicals Ltd.; Sydney–Melbourne; Methanol (CH_3_OH, B.N. 19758725, 67-56-1) from Scharlau, Barcelona, Spain.

Jarrah (*Eucalyptus marginata*) and Marri (*Corymbia calophylla*) pollen, both harvested in October 2019, were purchased from Davies Apiaries in Oldbury, 6121, WA, Australia. 

### 2.2. Extract Preparation

The bee pollen samples had already been dried, processed, and packed commercially, and no further treatment was undertaken prior to their analysis. To obtain pulverised samples (75–150 µm), the crude pollen grains were milled for 5 min using a commercial grinder (Breville Coffee Grinder Model BCG200). An amount of 0.5 g of pollen samples (crude, and non-pulverised) were separately extracted using ethanol, methanol, deionised water, and 70% ethanol in water, and the following extraction procedures:

*Agitation.* Agitation extraction was performed based on a protocol reported by Aleksieva et al. [72] with minor modifications. The extraction was carried out over 2 h in 7 mL of solvent using a hotplate magnetic stirrer (LLG Uni stirrer 3, John Morris Group) operating at a speed of 1500 rpm, with the temperature set at 40 °C. After 2 h, the solvent was decanted, and was replaced with fresh solvent and the extraction process was repeated two more times for the sample. 

*Maceration.* Maceration extraction was performed based on a protocol reported by Kaškonienė et al. [95] with minor modifications. The bee pollen sample was macerated in 7 mL of extraction solvent at room temperature (25 °C) at a speed of 160 rpm shaking (Memmert Shaker Bath, Model WNB 22) over three days and solvent changes every 24 h. 

*Reflux.* Reflux extraction was performed using an Electromantle reflux set-up and the protocol reported by Cheng et al. [81] with slight modifications. The method employed 21 mL of solvent and an extraction temperature determined by the boiling point of the respective solvent. Extraction was performed once over 2 h for each sample.

*Sonication.* Sonication extraction was performed following a procedure developed by Yan et al. [79] with slight modifications. The extraction was performed with 21 mL of solvent using a probe sonicator (Sonics Vibra Cell Model VCX130) operating at 130 Watts and 20 kHz. The amplitude was set at 100% and the extraction process was carried out once for 30 min on an ice bath.

Following extraction, the solvent was filtered (Whatman #4 filter paper) and, if the extraction process was repeated, filtrates were combined, and made up to 25 mL with the respective solvent. The resulting solutions were stored at −85 °C until further analysis. Every extraction process was carried out in triplicate for each pollen sample, and the responses combined to determine the mean. Table 2 summarises the independent variables studied along with their corresponding abbreviations.

### 2.3. Determination of Total Phenolic Content

A sugar solution was used as a blank in order to account for the potential sugar matrix interference in the assay. The sugar solution was prepared by diluting 2 g of a sugar stock solution (21.625 g of fructose, 18.125 g of glucose, 1.000 g of maltose, 0.750 g of sucrose and 8.500 g of water) to 5 mL (40%) with deionised water. The solution was stored under refrigeration and used within a week. A dilute Folin–Ciocalteu reagent was prepared by mixing 1 mL of Folin–Ciocalteu reagent with 30 mL deionised water. A 0.75% anhydrous sodium carbonate solution was prepared by mixing 0.1875 g Na_2_CO_3_ in 25 mL water. A 2 mg/mL gallic acid stock solution was prepared by dissolving 200 mg of gallic acid in 100 mL deionised water and standards ranging in concentration from 0.18 mg/mL to 0.06 mg/mL were prepared by diluting the stock with water. 

The TPC assay was performed based on the methodology described by Liberato et al. [116] with minor modifications. In brief, for the analysis, 200 µL of pollen extract or 100 µL of gallic acid standard spiked with 100 µL of sugar solution were placed in a test tube followed by the addition of 1 mL of the diluted Folin–Ciocalteu reagent. The mixture was allowed to react for 5 min before 800 µL of Na_2_CO_3_ was added. The mixture was kept in the dark for 2 h before the absorbance was measured at 760 nm (Carry 60 Bio UV–Vis spectrophotometer) using 100 µL of water spiked with 100 µL of sugar solution along with other TPC reagents as a blank. The analysis was carried out in triplicate and the mean results for each sample were obtained. The antioxidant activity was expressed as mg of gallic acid equivalent (GAE) per g of pollen.

(1) $$\mathrm{TPC}\;\mathrm{Value}\;\mathrm{of}\;\mathrm{Sample}\;\left(\mathrm{mg}\right)\mathrm{Gallic}\;\mathrm{Acid}\;=\frac{\left(\Delta \mathrm{Abs}-\mathrm{intercept}\right)}{\mathrm{slope}}\times \;\mathrm{Dilution}\;\mathrm{Factor}$$

### 2.4. Determination of Antioxidant Activity using Ferric Reducing Antioxidant Power (FRAP) Assay

The FRAP reagent was prepared by mixing in proportions of 1:1:10 (*v*/*v*/*v*) 10 mM TPTZ (0.31 g dissolved in 100 mL of 40 mM HCl), 20 mM FeCl_3_·6H_2_O (0.5406 g dissolved in 100 mL of deionised water) and 300 mM acetate buffer pH 3.6 (3.1 g C_2_H_3_NaO_2_, 16.00 mL of glacial acetic acid dissolved in 1000 mL of deionised water). The reagent was freshly prepared and incubated at 37 °C prior to each test. For the standard curve, a 2 mM stock solution of FeSO_4_·7H_2_O was prepared by dissolving 55.6 mg of FeSO_4_·7H_2_O in 100 mL of deionised water. Standards ranging in concentration from 1200 µM to 200 µM were prepared prior to each experiment, stored on ice, and used within 2 h. The standard at 600 µM was used as a positive control in each experiment. 

The FRAP assay, which is based on the reduction of ferric 2,4,6-tris(2-pyridyl)-1,3,5-triazine [Fe(III)-TPTZ] to ferrous complex at low pH followed by a spectrophotometric analysis, was performed according to the protocol described by Almeida et al. [60] with minor modifications. In brief, 20 µL of pollen extract or standards were mixed with 180 µL of FRAP reagent in a 96-well microplate, incubated at 37 °C for 30 min and the absorbance of the reaction mixture was determined at 620 nm (BMG Labtech POLARstar Optima Microplate Reader). The mean of triplicate analysis results was calculated and the FRAP activity was determined on the interpolation of the standard curve and expressed as µmol Fe^2+^ equivalent (Fe^+2^ E)/g FW of pollen.

(2) $$\mathrm{FRAP}\;\mathrm{Value}\;\mathrm{of}\;\mathrm{Sample}\;\left(µM\right)\mathrm{Fe}\;\left(\mathrm{II}\right)=\frac{\left(\Delta \mathrm{Abs}-\mathrm{intercept}\right)}{\mathrm{slope}}\times \;\mathrm{Dilution}\;\mathrm{Factor}$$

### 2.5. Determination of Antioxidant Activity using the 2,2-diphenyl-1-picrylhydrazyl (DPPH) Radical Scavenging Assay 

This colorimetric assay utilises 2,2-diphenyl-1-picrylhydrazyl (DPPH) radicals, which are purple in colour; the colour decays in the presence of antioxidant agents, seen in a change in absorbance at 517 nm. The DPPH reaction mixture was made up of 0.130 mM DPPH reagent (5.1262 mg of DPPH in 100 mL of methanol), 100 mM NaC_2_H_3_O_2_ buffer pH 5.5 (7.355 g of NaC_2_H_3_O_2_ and 0.621 g of HC_2_H_3_O_2_) and bee pollen extract. Trolox in a concentration range of 600–100 µM (aqueous, pH adjusted to pH 7.0 to completely solubilize in water) was used as the standard, with the 400 µM standard acting as a positive control throughout all tests. 

The DPPH assay adopted in this experiment is based on the protocol described by Karabagias et al. [117] with minor modifications. In brief, 10 µL of bee pollen extract or Trolox standards were placed in a 96-well microplate, followed by the addition of 100 µL of NaC_2_H_3_O_2_ buffer and 190 µL of 0.130 mM methanolic DPPH solution. The reaction mixture was kept in the dark for 120 min before the absorbance was measured at 520 nm using the microplate reader. The mean radical scavenging activity of triplicate samples was expressed as the Trolox equivalent (TE), calculated by a linear regression analysis, and then expressed as µmol Trolox equivalent per g of pollen.

(3) $$\mathrm{DPPH}\;\mathrm{Value}\;\mathrm{of}\;\mathrm{Sample}\;\left(µM\right)\;\mathrm{Trolox}=\frac{\left(\Delta \mathrm{Abs}-\mathrm{intercept}\right)}{\mathrm{slope}}\times \;\mathrm{Dilution}\;\mathrm{Factor}$$

### 2.6. Statistical Analysis

Statistical analysis of the data was carried out using Design Expert 12 (StatEase Inc., Minneapolis, MN, USA) and two-way t-test or one-way ANOVA was analysed using Graphpad Prism 9 (GraphPad Software, San Diego, CA, USA). Pareto plots of regression coefficients of the independent variables were generated using Microsoft Excel. The signal to noise ratio was set at 5 times the standard deviation of observations for each response (*n* = 3). The model was developed based on the regression analysis of the statistical significance of variables, and the model coefficient was significant when the *F* value was larger than the critical *F* value (*p* < 0.05). The relative influence of factors was identified by comparing the magnitude of regression coefficients. Correlations between responses were established using Spearman regression analysis. When significant interactions between factors were identified, a two-way *t*-test or a one-way analysis of variance with Tukey’s post hoc comparison was used to identify differences between the groups, and the statistical significance was set at *p* > 0.05. 

## 3. Results

A thorough literature review of 101 published articles on the most widely used conditions for extracting antioxidant principles from bee pollen (Table 1) indicated a lack of a consistent approach. Researchers used a variety of antioxidant assays and analysis standards, and also, the manner by which the results were expressed varied greatly, making it difficult to conduct comparative analyses of findings across research groups. Furthermore, there appears to be no agreed protocol to guide the extraction process itself, which is required to establish baseline data for bee pollen of Australian origin in order to compare their antioxidant activity with other bee pollen samples. The gaps in information form the basis for this study, which aimed to optimise the extraction conditions for bee pollen collected in western Australia to enable the maximum extraction of antioxidant constituents as measured by the TPC, DPPH and FRAP assays. The Design of Experiment approach examined three independent variables: sample pulverisation, extraction solvent, and the extraction process. The two pollen types provided sample diversity to enable the development of a generalised extraction protocol that may be adopted for all types of bee pollen collected in western Australian and beyond. 

### 3.1. Analysis of the Optimisation Process

A multilevel factorial design was implemented using Design Expert 12 (StatEase Inc., Minneapolis, MN, USA), with sample pulverisation, extraction solvent, and the extraction process as independent variables. The conditions selected for each variable were based on the popularity of use, as reflected in the literature review (Table 1). The responses (dependent variables) measured were the TPC, DPPH, and FRAP antioxidant activities. As summarised in Table 3 (the full data set is available as Supplementary Material, Table S1), the extraction variables for the multilevel factorial design (categorical) were selected at two levels for pulverisation (A, crude = −, pulverised = +), four levels for extraction solvent (B, 70% ethanol: 30% = E70:30, ethanol = EtOH, water = H_2_O, and methanol = MtOH), and four levels for the extraction process (C, agitation = A, maceration = M, reflux = R, and sonication = S). The total runs consisted of 32 experimental points, and each point was triplicated. The sequence of the experiments was randomised, where the random numbers were generated by the Design Expert 12 software.

Pulverisation (Variable A) did not show any significant effect on the TPC, DPPH and FRAP data for the bee pollen (*p* value > 0.05) and was therefore removed from further analysis. Both solvent type (Variable B) and the extraction process (Variable C) were found to have significant impacts on the TPC, DPPH and FRAP antioxidant activity of bee pollen (*p* value < 0.05). A significant interaction between the solvent type and extraction process (Variables B and C) was observed for the TPC and FRAP responses (*p* value < 0.05), whereas the interaction of these variables did not influence the DPPH antioxidant activity.

**Figure 1:**
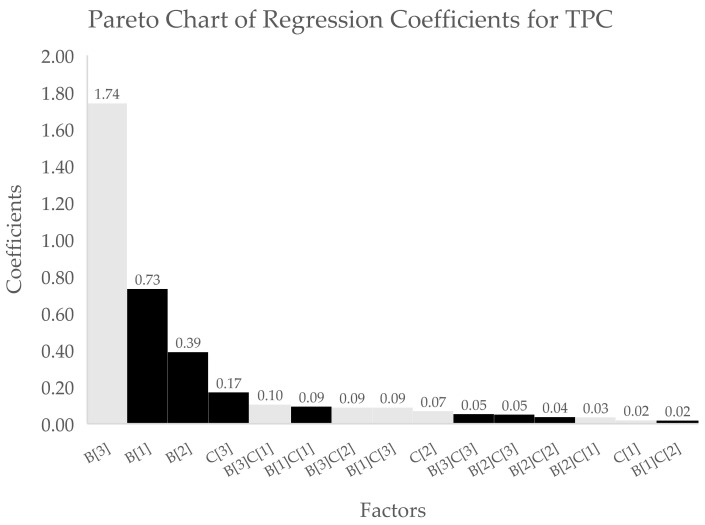
Pareto chart of regression coefficients for TPC assay data. X-axis indicates the different factors (B[*n*] = solvent type, C[*n*] = extraction process, and B[*n*]C[*n*] = interaction of solvent type and extraction process) while the values in the left y-axis indicate the coefficient values for the corresponding factors. Black bars indicate positive effect, while white bars indicate negative effect on TPC.

**Figure 2:**
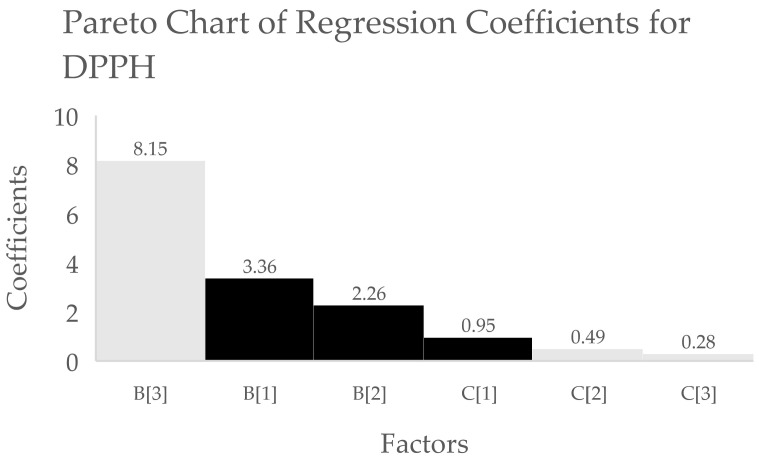
Pareto chart of regression coefficients for DPPH antioxidant activity data. X-axis indicates the different factors (B[*n*] = solvent type and C[*n*] = extraction process,) while the values in the left y-axis indicate the coefficient values for the corresponding factors. Black bars indicate positive effect, while white bars indicate negative effect on DPPH antioxidant activity.

**Figure 3:**
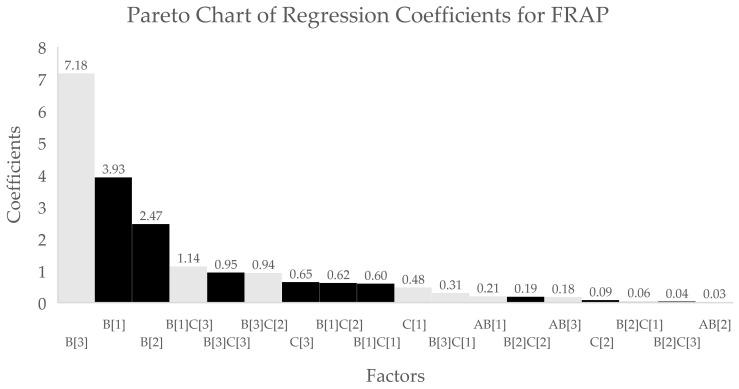
Pareto chart of regression coefficients for FRAP antioxidant activity data. X-axis indicates the different factors (A[*n*] = pulverisation, B[*n*] = solvent type, C[*n*] = extraction process, AB[*n*] = interaction of pulverisation and solvent, B[*n*]C[*n*] = interaction of solvent type and extraction process) while the values in the left x-axis indicate the coefficient values for the corresponding factors. Black bars indicate positive effect, while white bars indicate negative effect on DPPH antioxidant activity.

However, the larger coefficients obtained for extraction solvent relative to those for the extraction process indicate that the selection of solvent has a dominant effect on all three responses based on the tabulated regression coefficients (see Table 4), also seen in Pareto charts (Figure 1, Figure 2 and Figure 3). 

**Figure 4:**
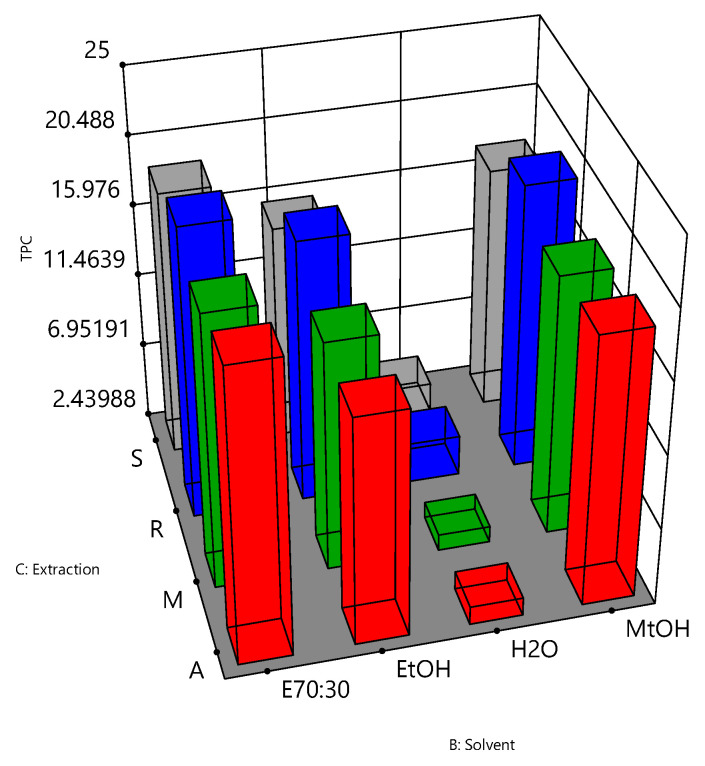
Interactive effects of extraction solvent and extraction method on the TPC assay for bee pollen samples. TPC values were expressed as mg gallic acid equivalent per gram pollen, B: extraction solvent (70% ethanol:30% = E70:30, ethanol = EtOH, water = H_2_O, and methanol = MtOH) and C: extraction process (agitation = A, maceration = M, reflux = R, and sonication = S).

**Figure 5:**
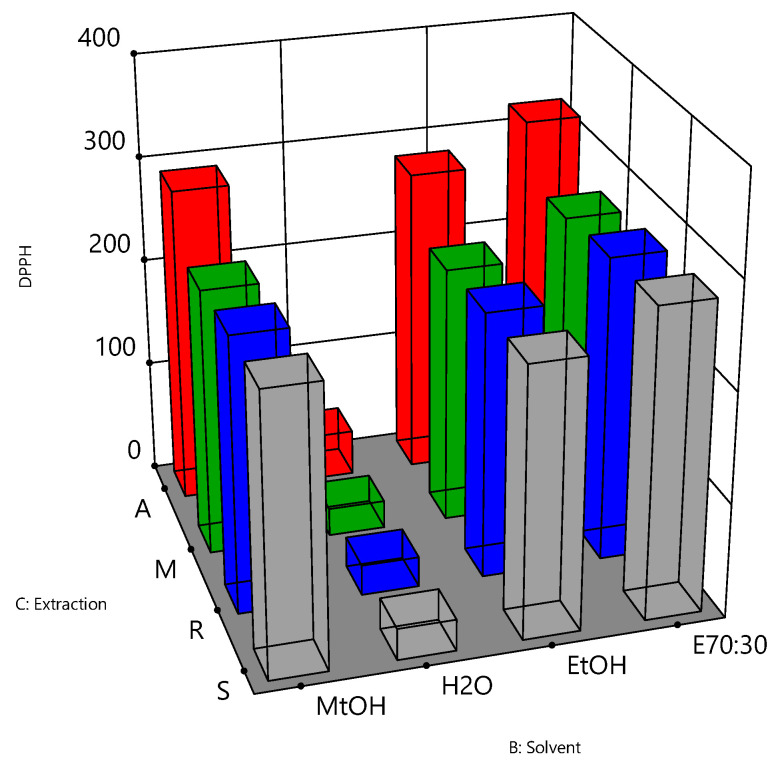
Effects of extraction solvent and extraction method on the DPPH antioxidant activity for bee pollen samples. DPPH values were expressed as µmol Trolox equivalent per gram pollen, B: extraction solvent (70% ethanol:30% =E70:30, ethanol = EtOH, water = H_2_O, and methanol = MtOH) and C: extraction process (agitation = A, maceration = M, reflux = R, and sonication = S).

**Figure 6:**
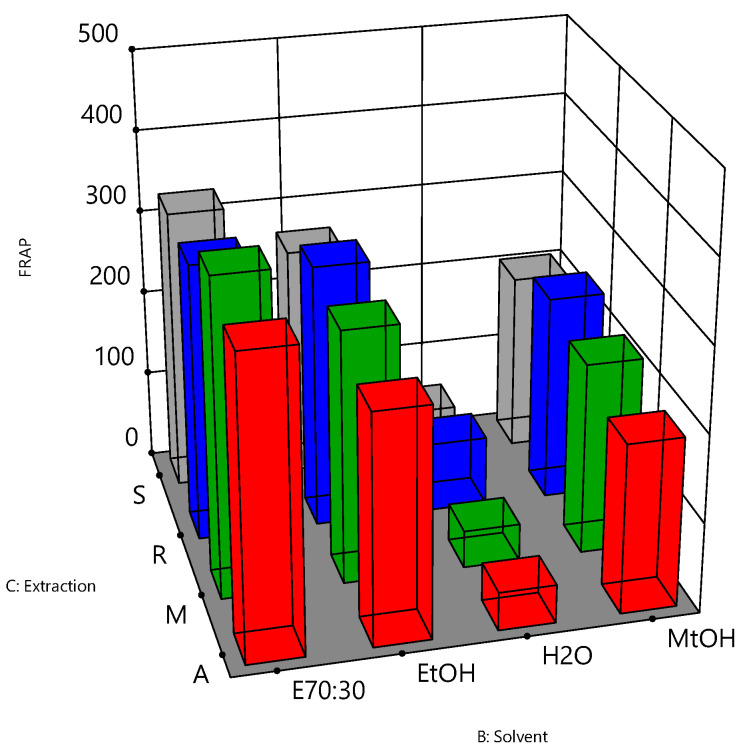
Interaction effects of extraction solvent and extraction method on the FRAP antioxidant activity for bee pollen samples. FRAP values were expressed as µmol Fe^+2^ equivalent per gram pollen, B: extraction solvent (70% ethanol:30% = E70:30, ethanol = EtOH, water = H_2_O, and methanol = MtOH) and C: extraction process (agitation = A, maceration = M, reflux = R, and sonication = S).

The relationships between the independent variables and TPC, DPPH and FRAP assays are further illustrated in three-dimensional graphs (Figure 4, Figure 5 and Figure 6). Figure 4 shows the results for the TPC assay of the bee pollen samples. The highest responses were observed when a solvent of 70% ethanol (E70:30) was coupled with reflux (R) or agitation (A) as the extraction process (*p* value < 0.05). These conditions may therefore represent optimal parameters for the extraction of the bee pollen samples for the TPC assay. For the DPPH antioxidant activity (Figure 5), the extraction solvent of 70% ethanol (E70:30) coupled with agitation (A) were the best extracting conditions for the bee pollen samples (*p* value < 0.001). Figure 6 shows that the extraction solvent of 70% ethanol (E70:30) coupled with maceration (M) produced the highest FRAP activity (*p* value < 0.05), and may therefore be considered as the combination of solvent type and the extraction process of choice for the FRAP assay of the bee pollen samples.

### 3.2. Correlation of TPC, DPPH and FRAP Antioxidant Activity

Bee pollen has been reported to contain many types of polyphenols [99] which are strongly correlated to the antioxidant activity of bee pollen [9]. In this study, a strong positive correlation was observed between the TPC and DPPH data, TPC and FRAP data as well as the DPPH and FRAP data, with correlation values of *ρ* = 0.6925 (*p* < 0.001), *ρ* = 0.7295 (*p* < 0.001) and *ρ* = 0.6520 (*p* < 0.001), respectively. Thus, the presence of polyphenols, captured in the pollen’s TPC, appears to positively influence its antioxidant capacity expressed in the DPPH and FRAP assays. However, it needs to be acknowledged that there are other pollen constituents, such as carotenoids, that could also influence the antioxidant capacity [82,106].

### 3.3. Choosing Optimum Conditions

**Figure 7:**
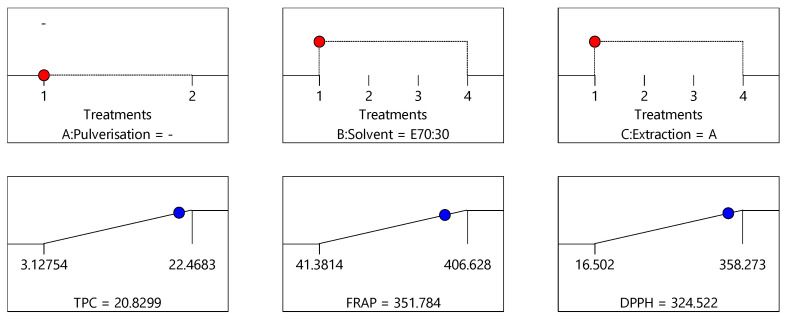
Proposed optimised extraction condition 1: crude/non-pulverised sample extracted using 70% ethanol:30% water (E70:30) and agitation (A), corresponding to the optimal conditions see in Figure 4 for TPC data, Figure 5 for DPPH data, and Figure 6 for FRAP data (desirability = 0.925).

**Figure 8:**
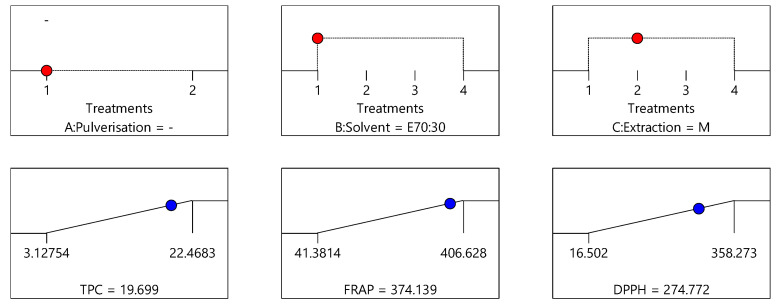
Proposed optimised extraction condition 2: crude/non-pulverised sample extracted using 70% ethanol:30% water (E70:30) and maceration (M), corresponding to the optimal conditions see in Figure 4 for TPC data, Figure 5 for DPPH data, and Figure 6 for FRAP data (desirability = 0.893).

**Figure 9:**
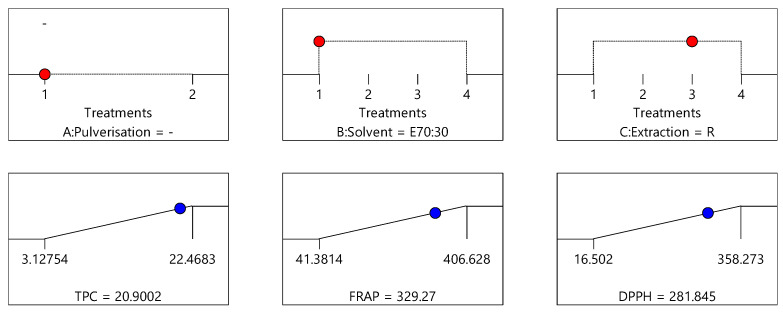
Proposed optimised extraction condition 3: crude/non-pulverised sample extracted using 70% ethanol:30% water (E70:30) and reflux (R), corresponding to the optimal conditions see in Figure 4 for TPC data, Figure 5 for DPPH data, and Figure 6 for FRAP data (desirability = 0.883).

The optimisation of the extraction process to maximise the three dependent variables was determined by employing a new variable desirability, which represents all responses simultaneously. Desirability is an objective function that is determined by ranked responses and its value ranges from zero to one, with one being most desirable. When there are several responses, the individual goals are combined to generate one desirability function, which was automated by Design Expert software 12 (StatEase, Inc. Minneapolis, MN, USA). The numerical optimisation based on the goal of the study finds a point that maximises the desirability function. The criteria adopted to determine the desirability function for this study are to maximise all responses. Figure 7, Figure 8 and Figure 9 provide predicted TPC, DPPH and FRAP values obtained for the three optimum conditions identified for the extraction of bee pollen samples.

Based on the model, the crude/non-pulverised pollen sample, extracted with 70% ethanol:30% water (E70:30) by agitation (A) as the extraction process produced the highest desirability (0.92) (Figure 7), followed by the crude/non-pulverised pollen sample, extracted with 70% ethanol:30% water (E70:30) by maceration (M) (desirability = 0.893) (Figure 8), and finally, the non-pulverised pollen sample, extracted with 70% ethanol:30% water (E70:30) by reflux (R) (desirability = 0.883) (Figure 9). These three conditions can therefore be considered to represent the optimal combination of sample pre-processing treatment, solvent type and extraction process to yield the maximum extraction of constituents from the bee pollen samples for the TPC, DPPH and FRAP assays. The bee pollen extracts prepared using the crude/non-pulverised pollen sample, extracted with 70% ethanol:30% water (E70:30) by agitation (A) as the extraction process were found to have an average total phenolic content of 20.86 mg GAE/g, DPPH antioxidant activity of 320.11 µmol TE/g and 342.28 µmol Fe^+2^ E/g FRAP activities, respectively. These values are very close to the predicted values of 20.83 mg GAE/g, 324.52 µmol TE/g and 351.78 µmol Fe^+2^ E/g, respectively, demonstrating the good fit of the chosen model.

## 4. Discussion

Commonly, the initial stage in studying the chemical composition and/or bioactivity of natural products, including bee pollen, includes a pre-extraction step, in which the material undergoes drying in order to preserve the biomolecules present in the sample [21]. This is often followed by grinding the dried material using a mortar and pestle, electric blender or various mills to decrease the particle size to enhance surface contact with the extraction solvent [21]. Particles that are too fine will, however, adsorb onto filters and impede filtration [118]. In this study, the effect of the pulverisation of bee pollen samples on its total phenolic content and associated antioxidant activity as captured by DPPH and FRAP assays was analysed. On the basis of these findings, the pulverisation process can be omitted from the extraction protocol.

The selection of solvent is crucial for solvent extraction, with selectivity for the target compounds, the target compound’s solubility as well as cost and safety to be considered. Based on the law of similarity and intermiscibility, solvents with a polarity value near the polarity of the solute are likely to perform better [118]. Water, along with a range of alcoholic and organic solvents such as methanol, ethanol, acetonitrile, acetone, hexane and diethyl ether are commonly utilised in the extraction of bioactive compounds [20]. The current study aimed to analyse the impact of different solvents, including water, methanol (M), ethanol (E) and the combination of ethanol and water at a ratio of 70:30 (*v*/*v*) (E70:30) on the total phenolic content as well as DPPH and FRAP antioxidant activity of pollen samples collected from western Australia. The findings of the study demonstrate that the extraction solvent had the strongest influence on the responses of the dependent variables (*p* <0.05). Among the solvents tested, extracts prepared with 70% ethanol:30% water (*v*/*v*) demonstrated the highest activity across all performed assays.

Based on the interactions observed in this study, it appears that TPC values are dependent on solvent type and the extraction process, and it can be concluded that TPC can be maximised when the pollen extraction is carried out either using 70% ethanol:30% water coupled with agitation or reflux. DPPH antioxidant activity is maximised following agitation as an extraction process in any of the investigated solvents, whereas FRAP antioxidant activity is highest when pollen is extracted with maceration coupled with 70% ethanol:30% water. Bee pollen contains 13–55% of carbohydrates [6,7,8] and it can be assumed that some can be carried over into the extract by polar solvents. Consequently, the prolonged heating of bee pollen at a high temperature might lead to the formation of Maillard products from these carbohydrates during processing [3]. Therefore, an extraction method that does not expose the pollen sample to prolonged high temperatures can be considered favourable. Maceration is an easy process in extracting antioxidant principles; however, it takes 72 h to complete, as compared to agitation, which only requires 6 h. Reflux is a very promising method of extraction, as it only requires 2 h to perform; however, depending on the chosen solvent, it might require high temperature in order to operate. Thus, in this light, agitation can be recommended as the optimal extraction process to maximise antioxidant principles obtained from bee pollen samples.

The term ‘phenolic’ or ‘polyphenol’ is chemically defined as a substance that possesses an aromatic ring bearing one or more hydroxyl substituents, including functional derivatives such as esters, methyl esters and glycosides. These bioactive compounds are extensively found across the plant kingdom and are closely linked with the sensory and nutritional quality of fresh and processed plant foods, including bee products such as honey and bee pollen [9]. Keskin and Özkök reported various multifloral bee pollen samples from the Czech Republic to have a total phenolic content ranging from 15.2 mg to 22.73 mg GAE/g pollen [38], whereas Mayda, Özkök et al. reported TPC values of 26.69 ± 0.595 and 43.42 ± 0.779 4 mg GAE/g pollen [58]. TPC for multifloral bee pollen from Morocco was reported as 45.96 ± 0.51 mg GAE/g pollen [63] and samples obtained from Hungary had TPC values ranging from 9.15 ± 0.12 to 13.63 ± 0.11 mg GAE/g pollen [72]. TPC values (mg GAE/g pollen) for the two western Australian monofloral pollen samples investigated as part of this study following the optimised extraction protocol by using non-pulverised samples extracted with 70% ethanol:30% via agitation was 20.86 mg GAE/g. With this, the TPC value of the western Australian pollens are broadly within typical ranges found for a range of bee pollen samples from a wide geographical spread. It needs to be highlighted, though, that comparisons between data generated in different studies need to be treated with caution, as the chosen extraction condition (solvent and extraction method) will impact on the obtained TPC data. Furthermore, the method used in the analysis of TPC in this study utilised a Folin–Ciocalteu method in slightly basic medium (0.75% sodium carbonate) as compared to most researchers that used 7.5% sodium carbonate. This amendment to the common assay protocol was found to be necessary, since reducing sugars present in alcoholic and aqueous pollen extracts can also be reduced by the reagent, and thus, lead to an overestimation of TPC. [119]

The optimised extraction protocol was also used to assess the antioxidant activity of the two western Australian pollen samples investigated in this study. Despite relatively high correlations between FRAP, DPPH and TPC values found in this study, it can be argued that in vitro antioxidant capacity should not be determined by means of a single antioxidant test model because of the diverse types of antioxidant that might be present in the sample as well as the intricacy of the natural product matrix and the variety of free radical reaction mechanisms involved in oxidation. Complementary antioxidant assays might, therefore, produce richer data [78]. Thus, in this study, the antioxidant potential of bee pollen extracts was determined by means of two different radical scavenging assays, namely DPPH and FRAP.

Using the DPPH assay, Rocchetti and Castiglioni reported Magnolia and Lamium bee pollen from Italy to have antioxidant activities of 11.9 and 134.7 µmol TE/g pollen, respectively [78]. Mărghitaş et al. reported DPPH antioxidant activities ranging from 135 to 2814 µmol TE/g for various monofloral pollens from Romania [28] and Saral et al. found DPPH scavenging activities between 13.87 and 15.04 mg TE/g for multifloral pollen from Turkey [74]. DPPH data generated in this study for the western Australian pollen samples were 320.11 µmol (equivalent to 80.12 mg) TE/g following the optimised extraction protocol using non-pulverised pollen extracted with E70:30 by agitation. These findings are within the range of values reported by others.

Using the FRAP assay, Zuluaga-Domínguez et al. reported 87.2 ± 15.6 μmol Trolox/g for multifloral pollen from Colombia [37], whereas Saral et al. found a FRAP activity ranging from 8.69 to 84.89 μmol Fe^2+^E/g for multifloral bee pollen from Turkey [74]. In this study, following the optimised extraction protocol by using non-pulverised pollen extracted with E70:30 by agitation, FRAP antioxidant activity was found to be 342.28 μmol Fe^+2^ E/g, which is higher than the values reported by Saral et al. However, the comparison of FRAP values appears even more difficult, not only because the results are dependent on the chosen extraction conditions but also because the studies use different reference standards (Trolox or Fe^2+^) to express their results.

## 5. Conclusions

Based on a thorough review of the extant literature, a number of common bee pollen processing steps, solvents and extraction methods were identified, which can all impact on extraction efficiency and thus result in different TPC, DPPH and FRAP values. The study reports on an in-depth investigation into the optimisation of the most popular extraction conditions for maximum TPC, DPPH and FRAP antioxidant activity using two bee pollen samples from western Australia. The effects of pulverisation, the chosen solvent (70% aqueous ethanol, ethanol, methanol and water) as well as the adopted extraction process (agitation, maceration, reflux and sonication) were determined in order to optimise the extraction parameters. The study’s findings demonstrate that non-pulverised pollen extracted with 70% aqueous ethanol coupled with agitation as the extraction method constitutes the best conditions in order to maximise the extraction of phenolics and antioxidant principles in these bee pollen samples.

## Figures and Tables

**Table 1:** Summary of extraction parameters used to determine the antioxidant activity of bee pollen.

Author	Reference	Country	Botanical Origin	Pre-Extraction	Pulve-Risation	Extraction Method	Solvent	Volume of Solvent	Extraction Tempe-Rature	Mixing/ Power	Extraction Time	No. of Extractions	Post-Extraction	Antioxidant Assays
Daoud, Ibrahim et al., 2019	[22]	Algeria	*Inula viscosa*	Stored at 4 °C	None	Maceration	80% Methanol (Aqueous)	10 g w/100 mL	N.I.	N.I.	24 h	2	Dried in vacuo	TPC, TFC, β-carotene bleaching
Machado De-Melo, Estevinho et al., 2016	[23]	Brazil	multifloral	Stored at −20 °C, some batches were oven dried at 42 °C, another was vacuum lyophilised	Crushed and sieved through a 0.595 mm sieve	Agitation	70% Ethanol (Aqueous)	2 g w/ 25 mL	70 °C	105 rpm	30 min	N.I.	Volume was adjusted to 25 mL	TPC, DPPH, ORAC
Kalaycıoğlu, Kaygusuz et al., 2017	[7]	Turkey	Chestnuts, Buckwheat, Oak, multifloral	N.I.	Ground in mortar and pestle	Maceration	Water	0.1 g w/5 mL	80 °C	None	15 min	N.I.	Filtered using Whatman #41	TPC, DPPH
Alimoglu, Guzelmeric et al., 2021	[24]	Turkey	multifloral	Stored at 20 °C	None	Agitation and Sonication	70% Ethanol (Aqueous)	5 g w/50 mL	40 °C	100 rpm, N.I.	1 h and 15 min	N.I.	Filtered using filter paper, dried in vacuo	TPC, TFC, DPPH, CUPRAC, FRAP
Pascoal, Rodrigues et al., 2014	[25]	Porugal and Spain	multifloral	Stored at 20 °C	None	Maceration	Methanol	1:2 *w*/*v*	RT	None	72 h	2	Filtered using Whatman #4, dried in vacuo,	TPC, TFC, TBARS, DPPH
Morais, Moreira et al., 2011	[26]	Portugal	multifloral	Stored at −20 °C	None	Maceration	Methanol	1:2 *w*/*v*	RT	None	72 h	2	N.I.	TPC, DPPH, β-carotene bleaching
Leja, Mareczek et al., 2006	[27]	Poland	multifloral	Stored at −18 °C	None	Maceration	80% Methanol (Aqueous)	N.I.	N.I.	None	N.I.	N.I.	N.I.	TPC, TFC, anthocyanidins, phenylpropanoids, DPPH, TAA
Mărghitaş, Stanciu et al., 2009	[28]	Romania	multifloral	N.I.	None	Maceration and Sonication	Methanol	2 g w/15 mL	RT	None	1 h and 15 min	3	Dried in vacuo	TPC, TFC, DPPH, FRAP, ABTS
Thakur and Nanda 2021	[29]	India	Coconut, Coriander, Rapeseed, and multifloral	Stored at −18 °C	Ground (method not indicated)	Maceration and Sonication	85% Methanol (Aqueous)	0.15:1	RT	None	2 h and 30 min	N.I.	Centrifuged, and Dried in vacuo,	TPC, TFC, DPPH, FRAP, ABTS, MCA,
Machado De-Melo, Estevinho et al., 2018	[30]	Brazil	*Mimosa* *caesalpiniifolia*, *Eucalyptus* spp., Rubiaceae, *Astrocaryum* *aculeatissimum*, Fabaceae, *Cocos nucifera*, *M. verrucosa*, *Myrcia* spp., *Alternanthera* spp., Asteraceae, *Brassica* spp., and multifloral	Stored at −4 °C	None	Maceration	Methanol	1:2 *w*/*v*	RT	None	72 h	2	N.I.	TPC, TFC, DPPH, ORAC
Machado De-Melo, Estevinho et al., 2018	[30]	Brazil	*Mimosa* *caesalpiniifolia*, *Eucalyptus* spp., Rubiaceae, *Astrocaryum* *aculeatissimum*, Fabaceae, *Cocos nucifera*, *M. verrucosa*, *Myrcia* spp., *Alternanthera* spp., Asteraceae, *Brassica* spp. and multifloral	Stored at −4 °C	None	Agitation	70% Ethanol (Aqueous)	2 g/25 mL	70 °C	105 rpm	30 min	N.I.	N.I.	TPC, TFC, DPPH, ORAC
Kaškonienė, Adaškevičiūtė et al., 2020	[31]	Latvia and Lithuania	multifloral	Pasteurisation at 95 °C for 20 min and stored at 6–8 °C	None	Agitation	80% Methanol (Aqueous)	2 g/20 mL	RT	180 rpm	N.I.	N.I.	7–10 μm paper filter (Labbox), followed by a 0.22 μm polyvinylidene fluoride (PVDF) membrane filter, stored at 4 C.	TPC, TFC, DPPH
Wan Omar, Azhar et al., 2016	[32]	Malaysia	*L. terminate*	N.I.	None	Sonication	Methanol	10 g/25 mL	41 °C	N.I.	1 h	N.I.	Centrifuged, filtered using 0.2 mm filter	DPPH
Khongkarat, Ramadhan et al., 2020	[33]	Thailand	Sunflower (*Helianthus annuus* L.)	Dried at 40 °C and stored at 25 °C	None	Agitation	Methanol	140 g/800 mL	15 °C	100 rpm	18 h	N.I.	Centrifuged and dried in vacuo	DPPH
Zhang, Wang et al., 2015	[34]	China	Rape (*Brassica campestris* L.)	Vacuum dried at 50 °C and stored at −18 °C	Ground (method not indicated)	Maceration	Water	1:20, *w*/*v*	RT	None	24 h	N.I.	Centrifuged, reconstituted to 100 mL	TPC, TFC, ABTS, DPPH, Reducing power
Zhang, Wang et al., 2015	[34]	China	Rape (*Brassica campestris* L.)	Rape (*Brassica campestris* L.)	Ground (method not indicated)	Maceration	25% Ethanol (Aqueous)	1:20, *w*/*v*	RT	None	24 h	N.I.	Centrifuged, reconstituted to 100 mL	TPC, TFC, ABTS, DPPH, Reducing power
Zhang, Wang et al., 2015	[34]	China	Rape (*Brassica campestris* L.)	Vacuum dried at 50 °C and stored at −18 °C	Ground (method not indicated)	Maceration	50% Ethanol (Aqueous)	1:20, *w*/*v*	RT	None	24 h	N.I.	Centrifuged, reconstituted to 100 mL	TPC, TFC, ABTS, DPPH, Reducing power
Zhang, Wang et al., 2015	[34]	China	Rape (*Brassica campestris* L.)	Vacuum dried at 50 °C and stored at −18 °C	Ground (method not indicated)	Maceration	75% Ethanol (Aqueous)	1:20, *w*/*v*	RT	None	24 h	N.I.	Centrifuged, reconstituted to 100 mL	TPC, TFC, ABTS, DPPH, Reducing power
Zhang, Wang et al., 2015	[34]	China	Rape (*Brassica campestris* L.)	Vacuum dried at 50 °C and stored at −18 °C	Ground (method not indicated)	Maceration	Ethanol	1:20, *w*/*v*	RT	None	24 h	N.I.	Centrifuged, reconstituted to 100 mL	TPC, TFC, ABTS, DPPH, Reducing power
Hemmami, Ben Seghir et al., 2020	[35]	Algeria	multifloral	N.I.	Crushed in a commercial blender and homogenised.	Sonication	Methanol	0.2 g w/2 mL	RT	N.I.	30 min	N.I.	Centrifuged, filtered using Whatman # 1, dried in vacuo, stored in 4 °C in brown bottle	TPC, TFC, TAC
Izuta, Narahara et al., 2009	[36]	Japan and Spain	Jara pringosa (*Cistus ladanifer*) and Jara blanca (*Cistus albidus*) [Spain]	N.I.	None	N.I.	95% Ethanol (Aqueous)	N.I.	RT	N.I.	N.I.	N.I.	N.I.	DPPH
Zuluaga-Domínguez, Serrato-Bermudez et al., 2018	[37]	Colombia	*Hypochaeris radicata* and *Brassica* sp.	Stored at 2 °C	None	Maceration	96% Ethanol (Aqueous)	1 g w/30 mL	N.I.	N.I.	24 h	N.I.	Filtered using 3 hw filter paper and reconstituted to 100 mL	TPC, TFC, ABTS, FRAP
Keskin and Özkök 2020	[38]	Turkey	multifloral	Dried at different conditions	None	Agitation	Ethanol	3 g/20 mL	N.I.	N.I.	12 h	N.I.	Filtered and the final volume was completed to 30 mL	TPC
Vasconcelos, Duarte et al., 2017	[39]	Brazil	multifloral	N.I.	None	Agitation	70% Ethanol (Aqueous)	1 g/10 mL	70 °C	150 rpm	30 min	2	Centrifuged	TPC, TFC, DPPH, FRAP
Suriyatem, Auras et al., 2017	[40]	Thailand	Longan	Stored at 20 °C	None	Maceration	Methanol	1:2 (*w*/*v*).	RT	Shaken by hand twice a day	72 h	2	Filtered using Whatman #4 and dried in vacuo	TPC, DPPH, ABTS
Cosmulescu, Trandafir et al., 2015	[41]	Romania	Walnut (*Juglans regia* L.)	N.I.	None	Sonication and maceration	Methanol	1 g/10 mL	RT	N.I.	60 min and 24 h	1	Filtered using 0.45 µm membrane filter	TPC, TFC, DPPH
Bridi, Atala et al., 2019	[42]	Chile	*Brassica rapa* and *Eschscholzia californica*	Stored at −20 °C	None	Sonication	Ethanol	1 g/10 mL	−20 °C	N.I.	10 min	3	Centrifuged and filtered using Whatman No.1 and reconstituted to 50 mL	TPC, TFC, FRAP, ORAC
Lopes, Vasconcelos et al., 2019	[43]	Brazil	multifloral	Stored at 4 °C	None	Maceration	70% Ethanol (Aqueous)	1:5 (*m*/*v*)	RT	None	72 h (solvent renewal every 24 h)	3	Dried in vacuo	TPC, TFC, FRAP, DPPH, ABTS
Lopes, Vasconcelos et al., 2020	[44]	Brazil	multifloral	Stored at 4 °C	None	Maceration	70% Ethanol (Aqueous)	1: 5 (*m*/*v*)	RT	None	72 h (solvent renewal every 24 h)	3	Dried in vacuo, lyophilised	TPC, TFC, FRAP, DPPH, ABTS
Futui and Thongwai 2020	[45]	Thailand	multifloral	Stored at −20 °C	Powdered (method not indicated)	Maceration	Water	1:10	45 °C	None	3 h	3	Filtered, dried in vacuo, lyophilised	TPC, TFC, DPPH
Futui and Thongwai 2020	[45]	Thailand	multifloral	Stored at −20 °C	Powdered (method not indicated)	Maceration	95% Ethanol (Aqueous)	1:10	RT	None	72 h (solvent renewal every 24 h)	3	Filtered, dried in vacuo, lyophilised	TPC, TFC, DPPH
Futui and Thongwai 2020	[45]	Thailand	multifloral	Stored at −20 °C	Powdered (method not indicated)	Sonication	Water	1:10	RT	N.I.	30 min	2	Filtered, dried in vacuo, lyophilised	TPC, TFC, DPPH
Nurdianah, Ahmad Firdaus et al., 2016	[46]	Malaysia	multifloral	Stored at 4 °C	None	Maceration	Ethanol	10 g/100 mL	RT	N.I.	24 h	1	Filtered, dried in vacuo, lyophilised, 4	DPPH
Fatrcova-Sramkova, Nozkova et al., 2013	[47]	Slovak	Poppy (*Papaver somniferum* L.), Rape (*Brassica napus* subsp. *napus* L.), Sunflower (*Helianthus annuus* L.).	Stored at −18 °C	Homogenised	Maceration	90% Ethanol (Aqueous)	5 g/50 mL	70 °C	N.I.	30 min		N.I.	DPPH, Reducing Power
Sun, Guo et al., 2017	[48]	China	Rape	Defatted with hexane	None	Sonication	70% Methanol (Aqueous)	N.I	RT	N.I	60 min		N.I.	TPC, TFC, DPPH, FRAP, ABTS
Borycka, Grabek-Lejko et al., 2016	[10]	Poland	multifloral	N.I.	Ground using mortar and pestle	Agitation	Water	2 g/15 mL	70 °C	N.I.	30 min		Filtered using Whatman #1 and dried in vacuo	TPC, TFC, DPPH, FRAP, ABTS,
Borycka, Grabek-Lejko et al., 2016	[10]	Poland	multifloral	N.I.	Ground using mortar and pestle	Agitation	70% Ethanol (Aqueous)	2 g/15 mL	70 °C	N.I.	30 min		Filtered usingWhatman #1 and dried in vacuo	TPC, TFC, DPPH, FRAP, ABTS
Borycka, Grabek-Lejko et al., 2016	[10]	Poland	multifloral	N.I.	Ground using mortar and pestle	Agitation	70% Methanol (Aqueous)	2 g/15 mL	70 °C	N.I.	30 min	1	Filtered, using Whatman #1 and dried in vacuo	TPC, TFC, DPPH, FRAP, ABTS
Su, Yang et al., 2020	[49]	China	Camellia, Rape, Rose, and Lotus	N.I.	Mechanical pulverisation	Sonication	Methanol	1:10	30 °C	100 W	1 h	1	Filtered, dried in vacuo	TPC, DPPH, RP, ABTS
Uçar, Barlak et al., 2017	[50]	Turkey	multifloral	N.I.	Ground (method not indicated)	Agitation	DMSO	5 g w/20 mL	60 °C	150 rpm	24 h	1	Centrifuged	TPC, TFC, FRAP, ABTS
Uçar, Barlak et al., 2017	[50]	Turkey	multifloral	N.I.	Ground (method not indicated)	Agitation	Water	5 g w/20 mL	60 °C	150 rpm	24 h	1	Centrifuged	TPC, TFC, FRAP, ABTS
Oroian, Ursachi et al., 2020	[51]	Romania	multifloral	Stored at −20 °C	None	Sonication	80% Methanol (Aqueous)	1:10–30	35 °C, 50 °C, 65 °C	100 W.	10–30 min	1	N.I.	TPC, TFC
Barbieri, Gabriele et al., 2020	[52]	Italy	multifloral	Stored at −20 °C	Powdered with mortar and pestle	Agitation	95% Ethanol (Aqueous)	50 mg/mL	RT	N.I.	1 h	N.I.	N.I.	TPC, TFC, FRAP
Shen, Geng et al., 2019	[53]	China	*Schisandra chinensis*	Dried at 37 °C	Pulverised (method not indicated)	Refluxed	70% Ethanol (Aqueous)	1:15	Boling Point	None	2 h	N.I.	Centrifuged, dried in vacuo, freeze dried	ABTS, FRAP
Saral, Yildiz et al., 2016	[54]	Turkey	*Castanea sativa* L.	N.I.	None	Maceration and sonication	Methanol	5 g/100 mL	RT	N.I.	24 h, 3 h	N.I.	Filtered, fried in vacuo	TPC, FRAP, DPPH
Pérez-Pérez, Vit et al., 2012	[55]	Venezuela	multifloral	N.I.	Ground in a mortar, frozen	homogenised	Water	0.1 g/5 mL	4 °C	N.I.	N.I.	N.I.	Centrifuged	TPC, ABTS
Pérez-Pérez, Vit et al., 2012	[55]	Venezuela	multifloral	N.I.	Ground in a mortar, frozen	homogenised	Methanol	0.1 g/5 mL	4 °C	N.I.	N.I.	N.I.	Centrifuged	TPC, ABTS
Pérez-Pérez, Vit et al., 2012	[55]	Venezuela	multifloral	N.I.	Ground in a mortar, frozen	homogenised	95% Ethanol (Aqueous)	0.1 g/5 mL	4 °C	N.I.	N.I.	N.I.	Centrifuged	TPC, ABTS
Daudu 2019	[56]	Nigeria	multifloral	Oven dried at 40 °C	Ground (method not indicated)	Maceration	50% Ethanol (Aqueous)	0.5 g/5 mL	N.I.	N.I.	N.I.	N.I.	Filtered	TPC, TFC, NO2, DPPH, TAC, RP, Metal Chelating
Daudu 2019	[56]	Nigeria	multifloral	Oven dried at 40 °C	Ground (method not indicated)	Maceration	Methanol	0.5 g/5 mL	N.I.	N.I.	N.I.	N.I.	Filtered	TPC, TFC, NO2, DPPH, TAC, RP, Metal Chelating
Daudu 2019	[56]	Nigeria	multifloral	Oven dried at 40 °C	Ground (method not indicated)	Maceration	Water	0.5 g/5 mL	N.I.	N.I.	N.I.	N.I.	Filtered	TPC, TFC, NO2, DPPH, TAC, RP, Metal Chelating
Daudu 2019	[56]	Nigeria	multifloral	Oven dried at 40 °C	Ground (method not indicated)	Maceration	Ethanol	0.5 g/5 mL	N.I.	N.I.	N.I.	N.I.	Filtered	TPC, TFC, NO2, DPPH, TAC, RP, Metal Chelating
Stanciu, Dezmirean et al., 2016	[57]	Romania	multifloral	Stored at −18 °C	Ground (method not indicated)	N.I.	Hexane:dichloromethane 1:1	N.I.	N.I.	N.I.	1 h	N.I.	N.I.	TPC, Total carotenoid, ORAC
Stanciu, Dezmirean et al., 2016	[57]	Romania	multifloral	Stored at −18 °C	Ground (method not indicated)	N.I.	acetone:water:acetic acid 70:29.5:0.5	N.I.	N.I.	N.I.	1 h	N.I.	N.I.	TPC, Total carotenoid, ORAC
Mayda, Özkök et al., 2020	[58]	Turkey	multifloral	Stored at −18 °C	None	Agitation and sonication	95% Ethanol (Aqueous)	1.5 g/10 mL	40 °C	Vortex Mixer	60 min	N.I.	Filtered through 0.45 μm filter	TPC, TFC, DPPH, ABTS
Anjos, Fernandes et al., 2019	[59]	Portugal	multifloral	Stored at −18 °C	None	Agitation	80% Ethanol (Aqueous)	11 g/200 mL	RT	4× *g*	24 h	N.I.	Centrifuged, dried in vacuo, freeze dried	TPC, TFC, DPPH, RP
Almeida, Reis et al., 2017	[60]	Brazil	multifloral	N.I.	None	Agitation	80% Ethanol (Aqueous)	10 g/100 mL	40 °C	150 rpm	60 min	N.I.	Filtered, dried in vacuo, freeze dried	TPC, TFC, ABTS, DPPH, FRAP, Coupled oxidation of b-carotene and linoleic acid assay
Fatrcova-Sramkova, Nozkova et al., 2016	[61]	Slovakia	*Helianthus annuus*	Stored at 35 °C	Homogenised (method not indicated)	Maceration	90% Ethanol (Aqueous)	5 g/50 mL	70 °C	N.I.	30 min	N.I.	Stored at 5 °C	TPC, Caroteniods, RP, Flavonoids
Mosic, Trifkovic et al., 2019	[62]	Serbia	multifloral	N.I.	None	Sonication	70% Methanol (Aqueous)	1 g/10 mL	N.I.	N.I.	1 h	N.I.	N.I.	TPC
El Ghouizi, Menyiy et al., 2020	[63]	Morocco	multifloral	N.I.	None	Agitation	70% Ethanol (Aqueous)	2 g/15 mL	70 °C	N.I.	30 min	N.I	Filtered using Whatman #5	TPC, TFC, TAC, RP
Rebiai and Lane 2012	[64]	Algeria	Carrot, Rosemary, Eucalyptus, and multifloral	Stored at 4 °C	Homogenised in blender	Soxhlet	Methanol	5 g w/175 mL	70 C	None	2 h	1	Dried in vacuo	TPC, TFC, TAC
Araujo, Chambo et al., 2017	[65]	Brazil	*Cocos nucifera*, *Miconia* spp., *Spondias* spp., *Myrcia* spp., *Eucalyptus* spp.	N.I.	None	Agitation	Methanol	1:1	N.I.	N.I.	24 h	3	Dried in vacuo	TPC, TFC, DPPH, A BTS
Barbara, Machado et al., 2015	[66]	Brazil	multifloral	N.I.	None	Agitation	Methanol	1:1	N.I.	N.I.	24 h	3	Dried in vacuo	TPC, TFC
Carpes, Mourao et al., 2009	[67]	Brazil	multifloral	Stored at −12 to −15 °C	Crushed using commercial blender	Maceration	70% Ethanol (Aqueous)	2 g/15 mL	70 °C	None	30 min	N.I.	Filtered and stored	TPC, TFC, DPPH
Sartini, Djide et al., 2019	[68]	Indonesia	multifloral	N.I.	Coarse powder (method not indicated)	Maceration	80% Ethanol (Aqueous)	100 g/1 L	RT	None	120 h	N.I.	Dried in vacuo, freeze dried	TPC, TFC, DPPH
Le Blanc, Davis et al., 2009	[1]	USA	Mesquite, Yucca, Palm, Terpentine Bush, Mimosa and Chenopod	N.I.	None	Sonication	Water	50 mg/mL	41 °C	None	90 min	N.I.	N.I.	TPC, Total flavones and flavonol, total flavonones, DPPH, FRAP
Le Blanc, Davis et al., 2009	[1]	USA	Mesquite, Yucca, Palm, Terpentine Bush, Mimosa and Chenopod	N.I.	None	Sonication	Methanol	50 mg/mL	41 °C	None	90 min	N.I.	N.I.	TPC, Total flavones and flavonol, total flavonones, DPPH, FRAP
Le Blanc, Davis et al., 2009	[1]	USA	Mesquite, Yucca, Palm, Terpentine Bush, Mimosa and Chenopod	N.I.	None	Sonication	Ethanol	50 mg/mL	41 °C	None	90 min	N.I.	N.I.	TPC, Total flavones and flavonol, total flavonones, DPPH, FRAP
Le Blanc, Davis et al., 2009	[1]	USA	Mesquite, Yucca, Palm, Terpentine Bush, Mimosa and Chenopod	N.I.	None	Sonication	Propanol	50 mg/mL	41 °C	None	90 min	N.I.	N.I.	TPC, Total flavones and flavonol, total flavonones, DPPH, FRAP
Le Blanc, Davis et al., 2009	[1]	USA	Mesquite, Yucca, Palm, Terpentine Bush, Mimosa and Chenopod	N.I.	None	Sonication	2-propanol	50 mg/mL	41 °C	None	90 min	N.I.	N.I.	TPC, Total flavones and flavonol, total flavonones, DPPH, FRAP
Le Blanc, Davis et al., 2009	[1]	USA	Mesquite, Yucca, Palm, Terpentine Bush, Mimosa and Chenopod	N.I.	None	Sonication	Acetone	50 mg/mL	41 °C	None	90 min	N.I.	N.I.	TPC, Total flavones and flavonol, total flavonones, DPPH, FRAP
Le Blanc, Davis et al., 2009	[1]	USA	Mesquite, Yucca, Palm, Terpentine Bush, Mimosa and Chenopod	N.I.	None	Sonication	DMF	50 mg/mL	41 °C	None	90 min	N.I.	N.I.	TPC, Total flavones and flavonol, total flavonones, DPPH, FRAP
Le Blanc, Davis et al., 2009	[1]	USA	Mesquite, Yucca, Palm, Terpentine Bush, Mimosa and Chenopod	N.I.	None	Sonication	ACN	50 mg/mL	41 °C	None	90 min	N.I.	N.I.	TPC, Total flavones and flavonol, total flavonones, DPPH, FRAP
Ceksteryte, Kurtinaitiene et al., 2016	[12]	Lithuania	multifloral	Stored at 5–8 °C	None	Agitation	80% Methanol (Aqueous)	6 g/10 mL	N.I.	None	5 min	N.I.	Centrifuged, dried in vacuo, freeze dried	TPC, DPPH, ABTS, ORAC
Özcan, Aljuhaimi et al., 2019	[69]	Brazil	multifloral	Stored at −18 °C	None	Sonication	Methanol	0.5 g/12 mL	N.I.	None	10 min	N.I.	Centrifuged, dried in vacuo	TPC, DPPH, Carotenoid, Minerals
Zuluaga-Dominguez and Quicazan 2019	[70]	Colombia	*Hypochaeris* spp., and *Brassica* spp.	N.I.	None	Agitation	96% Ethanol (Aqueous)	1 g/30 mL	N.I.	N.I.	24 h	N.I.	Filtered, volume completed quantitatively to 100 mL	TPC, ABTS, FRAP
Duran A 2019	[71]	Colombia	multifloral	N.I.	None.	Agitation	96% Ethanol (Aqueous)	1 g/30 mL	N.I.	N.I.	24 h	N.I.	Filtered, volume completed quantitatively to 100 mL	TPC, TEAC, FRAP
Aleksieva, Mladenova et al., 2021	[72]	Bulgaria	multifloral	Lyophilised	None	Agitation	Ethanol	0.5 g/7.5 mL	RT	N.I.	2 h	N.I.	filtered	TPC, TFC, DPPH, TEAC
Aleksieva, Mladenova et al., 2021	[72]	Bulgaria	multifloral	Lyophilised	None	Agitation	Water	0.5 g/7.5 mL	RT	N.I.	2 h	N.I.	filtered	TPC, TFC, DPPH, TEAC
Atsalakis, Chinou et al., 2017	[73],	Greece	*Cistus creticus*	Stored at −20 °C	None	Maceration	Cyclohexane, Dichloromethane, Butanol and Water	27.5 g/150 mL	N.I.	None	N.I.	N.I.	N.I.	TPC, TFC, DPPH, ABTS
Saral, KiliÇArslan et al., 2019	[74]	Turkey	multifloral	Stored at 4 °C	Blended	Maceration	Methanol	N.I.	RT	None	24 h	N.I.	Filtered using Whatman filter paper #4 and then stored at 4 °C	TPC, TFC, CUPRAC, FRAP, DPPH
Al-Salem, Al-Yousef et al., 2020	[75]	Saudi Arabia	multifloral	N.I.	None	Maceration	95% Ethanol (Aqueous)	N.I.	RT	None	48 h	N.I.	Filtered, dried in vacuo	Catalase (CAT) assay, Vitamin C (ascorbic acid) assay, Glutathione (GSH) assay, Glutathione S-Transferase (GST) activity
Gabriele, Parri et al., 2015	[76]	Italy	multifloral	Stored at −20 °C	None	Agitation	95% Ethanol (Aqueous)	N.I	RT	N.I.	1 h	N.I	Filtered, stored at 4°C	TPC, TFC, DPPH, ORAC
Yildiz, Can et al., 2013	[77]	Turkey	multifloral	Dried in 40 °C	Powder (method not indicated)	Sonication	Methanol	1 g/10 mL	N.I.	None	3 h	N.I.	Filtered	TPC, TFC, Total Anthocyanins, Total Carotenoids, FRAP, DPPH
Rocchetti, Castiglioni et al., 2019	[78]	Italy	multifloral	Stored in the dark at room temperature	Ground (method not indicated)	Agitation	70% Methanol (Aqueous)	0.5 g/5 mL	N.I.	Shaking	5 min	N.I	Stored at −20 °C	TPC, DPPH, ABTS, ORAC
Yan, Li et al., 2019	[79]	China	*Brassica campestris* L.	N.I.	Superfine jet pulverisation Combined low-pressure jet-boiling device	Sonication	80% Acetone (Aqueous)	2 g/15 mL	N.I.	None	30 min	N.I.	Freeze dried and stored at −40 °C	TPC
Rebiai and Lanez 2013	[80]	Algeria	multifloral	N.I.	None	Maceration	Methanol	5 g/50 mL	RT	None	24 h	3	Filtered, refrigerated	TPC, TFC, cyclic voltammetry techniques
Cheng, Chen et al., 2019	[81]	China	multifloral	N.I.	None	Reflux	75% Ethanol (Aqueous)	1:10	75 °C	None	2 h	2	Centrifuged, dried in vacuo,	DPPH, FRAP
Muñoz, Velásquez et al., 2020	[82]	Chile	*Brassica campestris* and *Galega officinalis*	Stored at −18 °C	None	Sonication	Methanol	1 g/7.5 mL	N.I.	50 Hz.	30 min	1	Centrifuged, filtered at 0.45 um, refrigerated	TPC, TFC, FRAP
Yesiltas, Capanoglu et al., 2015	[83]	Turkey and Spain	multifloral	Stored at −18 °C	Ground (method not indicated)	Maceration and sonication	Methanol	2 g/15 mL	RT	N.I.	3 d, 15 min	1	Centrifuged	TPC, TFC, ABTS, FRAP, DPPH, CUPRAC
Mejias and Montenegro 2012	[84]	Chile	multifloral	N.I.	None	Suspension	Water	N.I.	N.I.	None	N.I.	N.I.	N.I.	TPC, DPPH, FRAP
Negri, Teixeira et al., 2011	[85]	Brazil	multifloral	Stored at −18 °C	None	Maceration	70% Methanol (Aqueous)	1.0 g/75 mL	RT	N.I.	45 min	N.I.	Filtered, volume completed quantitatively to 100 mL	TPC, DPPH
Campos, Webby et al., 2003	[86]	New Zealand, Portugal	*Salix atrocinera* Brot., *Ranunculus sardous* Crantz, and *Ulex europeus* L. (Portugal and New Zealand); *Eucalyptus globulus* Labill., *Cistus ladanifer* L., *Echium plantagineum* L., and *Erica australis* L. (Portugal); and *Metrosideros* *umbellata*, *Ixerba brexioides*, and *Knightia excelsa (New* *Zealand)*	N.I.	None	Sonication	50% Ethanol (Aqueous)	N.I.	N.I.	None	N.I.	N.I.	Centrifuged	DPPH
Ulusoy and Kolayli 2014	[87]	Turkey	multifloral	Stored at 4 °C	None	Sonication	Methanol	N.I.	RT	None	1 h	3	Filtered, dried in vacuo	TPC, FRAP, CUPRAC, DPPH
Mohdaly, Mahmoud et al., 2015	[88]	Egypt	Maize *(Zea mays)*	Stored at 4 °C	Powdered (method not indicated)	Maceration	Methanol	10.0 g/100 mL	RT	N.I.	12 h	1	Filtered (Whatman #1), dried in vacuo	DPPH, ABTS
De-Melo, Estevinho et al., 2018	[89]	Brazil	*Alternanthera*, *Anadenanthera*, *Cocos nucifera*, *Mimosa caesalpiniaefolia*, *Myrcia*, and *Mimosa scabrella*	Stored at 4 °C	Crushed using commercial blender	Maceration	70% Ethanol (Aqueous)	2 g/15 mL	70 °C	None	30 min	1	Filtered	TPC, TFC, DPPH
De-Melo, Estevinho et al., 2018	[89]	Brazil	*Alternanthera*, *Anadenanthera*, *Cocos nucifera*, *Mimosa caesalpiniaefolia*, *Myrcia*, and *Mimosa scabrella*	Stored at 4 °C	Crushed using commercial blender	Maceration	Methanol	2 g/15 mL	70°C	None	30 min	1	Filtered	TPC, TFC, DPPH
Sahin and Karkar 2019	[90]	Turkey	Chestnut	N.I.	None	Sonication	Ethanol	3 g/30 mL	65 °C	None	30 h	1	Filtered	TPC, FRAP, ABTS, CHROMAC
Belina-Aldemita, Schreiner et al., 2020	[91]	Philippines	multifloral	Under argon at −24 °C, defatted with hexane 3 times	Ground (method not indicated)	Macerated	Methanol	1 g/5 mL	RT	N.I.	1 h	3	Centrifuged, stored at −20 °C	TPC, TFC, and TMAC
Belina-Aldemita, Schreiner et al., 2020	[91]	Philippines	multifloral	Under argon at −24 °C, defatted with hexane 3 times	Ground (method not indicated)	Sonication	Methanol	1 g/5 mL	RT	N.I.	1 h	3	Centrifuged, stored at −20 °C	TPC, TFC, and TMAC
Belina-Aldemita, Schreiner et al., 2020	[91]	Philippines	multifloral	Under argon at −24 °C, defatted with hexane 3 times	Ground (method not indicated)	Maceration	acidified Methanol solution (methanol and 1 N hydrochloric acid 85:15 *v/v*).	1 g/5 mL	50 °C	N.I.	30 min	3	Centrifuged, stored at −20°C	TPC, TFC, and TMAC
Zuluaga-Domínguez, Castro- Mercado et al., 2019	[92]	Colombia	*Hypochaeris* spp., and *Brassica* spp.	Stored at 4 °C	None	Agitation	96% Ethanol (Aqueous)	1 g/30 mL	N.I.	Low speed	24 h	1	Filtered using 3 hw filter paper and volume completed quantitatively to 100 mL	TPC, TFC, Total Carotenoids, FRAP, ABTS
Bujang, Zakaria et al., 2021	[93]	Malaysia	Maize (*Zea mays* L)	Stored at −20 °C	Crushed	Sonication	70% Ethanol (Aqueous)	2 g/15 mL	RT	None	30 min	1	Centrifuged, filtered using Whatman #2	TPC, TFC, DPPH
Yang, Zhang et al., 2019	[16]	China	Rose	N.I.	Ball milled	Sonication	70% Ethanol (Aqueous)	50 mg/1 mL	25 °C	600 W and a frequency of 20 kHz	4 h	1	Filtered	TFC, DPPH, ORAC, ABTS, In Vivo antioxidant
Kaskoniene, Kaskonas et al., 2015	[94]	Lithuania	multifloral	N.I.	None	Maceration	85% Methanol (Aqueous)	N.I.	RT	N.I.	24 h	3	Filtered	TPC, TFC, DPPH
Kaškonienė, Katilevičiūtė et al., 2018	[95]	Lithuania	multifloral	N.I.	None	Maceration	85% Methanol (Aqueous)	N.I.	RT	N.I.	24 h	3	Filtered	TPC, TFC, DPPH
Khider, Elbanna et al., 2013	[96]	Egypt	Maize (*Zea mays*), clover (*Trifolium alexandrinum*), and Date palm (*Phoenix dactylifera*)	Stored at 4 °C	Powdered (method not indicated)	Maceration	Methanol	50 g/500 mL	RT	None	12 h	1	Filtered using Whatman paper # 5	DPPH
Khider, Elbanna et al., 2013	[96]	Egypt	Maize (*Zea mays*), clover (*Trifolium alexandrinum*), and date palm (*Phoenix dactylifera*)	Stored at 4 °C	Powdered (method not indicated)	Maceration	Hexane	50 g/500 mL	RT	None	12 h	1	Filtered using Whatman paper # 5	DPPH
Canale, Benelli et al., 2016	[97]	Italy	multifloral	Stored at −20 °C	None	Sonication and agitation	80% Methanol (Aqueous)	0.5 g/15 mL	N.I. and 4°C	None and N.I.	30 min and 30 min	2	Filtered through 0.45 µm filter	TPC, TFC, Rutin
Freire, Lins et al., 2012	[98]	Brazil.	multifloral	N.I.	None	Sonication	Ethanol	N.I.	N.I.	N.I.	N.I.	N.I.	Filtered, dried in vacuo	TPC, DPPH, ABTS, Fe Chelating
Kim, Jo et al., 2015	[99]	Korea	multifloral	Dried at 40 °C and then stored in a freezer until use	None	Maceration	80% Methanol (Aqueous)	N.I.	N.I.	N.I.	N.I.	2	N.I.	DPPH, TPC
Zhang, Yang et al., 2016	[100]	China	Rapeseed (*Brassica campestris* L.)	N.I.	Powdered (method not indicated)	Sonication	80% Ethanol (Aqueous)	50 mL/g	80 °C	40 kHz	30 min	1	Filtered through 0.45 µm	TPC, FRAP
Zhang, Liu et al., 2020	[101]	China	Rapeseed (*Brassica campestris* L.)	Extracted with petroleum ether to remove the lipids	Powdered (method not indicated)	Sonication	80% Ethanol (Aqueous)	50 mL/g	80 °C	40 kHz	30 min	1	Dried in vacuo, lyophilised	DPPH, ABTS, FRAP
Castagna, Benelli et al., 2020	[102]	Italy	Chestnut	N.I.	None	Sonication	80% Methanol (Aqueous)	N.I.	4 °C	N.I.	30 min	1	Centrifuged and filtered through 0.45 µm filter	TPC, TFC
Oyarzun, Andia et al., 2020	[103]	Chile	multifloral	N.I.	None	Sonication	Ethanol	1.0 g in 10 mL	RT	37 kHz frequency and 240 W	10 min	3	Centrifuged and filtered through Whatman # 1	FRAP, ORAC, TPC, TFC
Asmae, Nawal et al., 2021	[104]	Morocco	multifloral	Stored at −20 °C	None	Maceration	70% Ethanol (Aqueous)	1.0 g in 10 mL	RT	N.I.	1 week	1	Filtered through Whatman # 1	TPC, Flavones and Flavonols Content, TAC, DPPH, RP, ABTS
Feas, Vazquez-Tato et al., 2012	[6]	Portugal	multifloral	N.I.	Ground (method not indicated)	Sonication and maceration	Methanol	1:2	RT	N.I.	30 min and 2 days	1	Centrifuged, dried in vacuo	TPC, TFC, DPPH, β-Carotene Bleaching
Rodriguez-Gonzalez, Ortega-Toro et al., 2018	[105]	Colombia	multifloral	N.I.	None	Microwave Assisted Extraction	Ethanol	1 g/10 or 50 mL	Varies	1350 W of power and 60 Hz	6, 12 and 24 s	1	Filtered and stored in −20°C	TPC, ABTS, FRAP
Rodriguez-Gonzalez, Ortega-Toro et al., 2018	[105]	Colombia	multifloral	N.I.	None	Sonication	Ethanol	1 g/10 mL	N.I.	5 kHz frequency and 250 W	15 min	1	Filtered and stored in −20°C	TPC, ABTS, FRAP
Velasquez, Rodriguez et al., 2017	[106]	Chile	multifloral	N.I.	None	Sonication	Water	1:1	N.I.	N.I.	1 h	5	Filtered using Whatman #2 dried in vacuo and the dry extract was reconstituted to 10 mL, filtered (EDLAB CA syringe filter 0.45 µm) and stored at −20 °C.	Total Carotenoid, TPC, FRAP
Carpes, de Alencar et al., 2013	[107]	Brazil	multifloral	N.I.	None	Maceration	70% Ethanol (Aqueous)	1:1	70 °C	N.I.	30 min	N.I.	N.I.	TPC, TFC, DPPH, Antioxidant activity by the coupled oxidation of b-carotene and linoleic acid
Santa Bárbara, Moreira et al., 2020	[108]	Brazil	multifloral	Oven dried, freeze dried, fresh	None	Agitation	70% Ethanol (Aqueous)	10 g/50 mL	RT	N.I.	45 min	1	Filtered, Dried in vacuo	b-Carotene bleaching assay, FRAP, DPPH, TPC, TFC
Carpes, Begnini et al., 2007	[109]	Brazil	multifloral	N.I.	Milled	Agitation	40% Ethanol (Aqueous)	2.0 g/15 mL	70 °C	N.I.	30 min	2	Stored at 5 ºC	oxidation of -carotene and linoleic acid, TPC
Carpes, Begnini et al., 2007	[109]	Brazil	multifloral	N.I.	Milled	Agitation	50% Ethanol (Aqueous)	2.0 g/15 mL	70 °C	N.I.	30 min	2	Stored at 5 ºC	oxidation of -carotene and linoleic acid, TPC
Carpes, Begnini et al., 2007	[109]	Brazil	multifloral	N.I.	Milled	Agitation	60% Ethanol (Aqueous)	2.0 g/15 mL	70 °C	N.I.	30 min	2	Stored at 5 ºC	oxidation of -carotene and linoleic acid, TPC
Carpes, Begnini et al., 2007	[109]	Brazil	multifloral	N.I.	Milled	Agitation	70% Ethanol (Aqueous)	2.0 g/15 mL	70 °C	N.I.	30 min	2	Stored at 5 ºC	oxidation of -carotene and linoleic acid, TPC
Carpes, Begnini et al., 2007	[109]	Brazil	multifloral	N.I.	Milled	Agitation	80% Ethanol (Aqueous)	2.0 g/15 mL	70 °C	N.I.	30 min	2	Stored at 5 ºC	oxidation of -carotene and linoleic acid, TPC
Carpes, Begnini et al., 2007	[109]	Brazil	multifloral	N.I.	Milled	Agitation	90% Ethanol (Aqueous)	2.0 g/15 mL	70 °C	N.I.	30 min	2	Stored at 5 ºC	oxidation of -carotene and linoleic acid, TPC
Zou, Hu et al., 2020	[110]	South Korea, China	multifloral	N.I.	None	Maceration	70% Ethanol (Aqueous)	N.I.	RT	N.I.	48 h	3	Filtered, dried in vacuo	TPC, TFC, DPPH
Paradowska, Zielińska et al., 2017	[111]	Poland	Buckwheat, Oilseeed Rape	N.I.	None	Sonication	80% Methanol (Aqueous)	0.2/10 mL	25 °C	N.I.	N.I.	1	Filtered through a sintered glass filter funnel	TPC, TFC, DPPH, FRAP, ORAC
Yildiz, Karahalil et al., 2014	[112]	Turkey	multifloral	N.I.	None	Suspension	Water	N.I.	N.I.	N.I.	N.I.	N.I.	Filtered	TPC, FRAP
Silva, Camara et al., 2009	[113]	Brazil	multifloral	N.I.	None	Sonication	Ethanol	N.I.	N.I.	N.I.	N.I.	N.I.	Filtered, dried in vacuo	DPPH
Dai, Ding et al., 2013	[114]	China	Rape, Rose, Camellia, Herba leonuri and Schizandra	N.I.	None	Sonication	Ethanol	50 mg/10 mL	N.I.	N.I.	N.I.	N.I.	Filtered, completed to 10 mL	TPC, DPPH
Amalia, Diantini et al., 2020	[115]	Indonesia	multifloral	N.I.	None	Suspension	Water	1:10	N.I.	N.I.	N.I.	N.I.	Filtered using Whatman^®^ # 41 paper, freeze-dried	DPPH

Legend: N.I.—not indicated, w/—with, *w/v*—weight over volume, TPC—total phenolic content, TFC—total flavonoid content, DPPH—2,2-diphenyl-1-picryl-hydrazyl-hydrate assay, ORAC—oxygen radical absorbance capacity, CUPRAC—Cupric reducing antioxidant capacity, FRAP—ferric reducing antioxidant capacity, TBARS—Thiobarbituric acid reactive substances, TAA—total antioxidant activity, ABTS—2,2′-azino-bis(3-ethylbenzothiazoline-6-sulfonic acid) (ABTS^•+^) radical cation-based assay, MCA—metal chelating activity, TAC—total antioxidant capacity, RP—reducing power, CHROMAC—chromium reducing antioxidant capacity.

**Table 2:** Independent variables used in the Design of Experiments to optimise the extraction of bee pollen for antioxidant evaluation.

Factors	Tested Conditions	Abbreviation
Pulverisation	Crude, non-pulverised	−
	Pulverised	+
Solvent	70% Ethanol	E70:30
	Ethanol	EtOH
	Methanol	MtOH
	Water	H_2_O
Extraction Process	Agitation	A
	Maceration	M
	Reflux	R
	Sonication	S

**Table 3:** Multilevel factorial experimental design to optimise the extraction of antioxidant components in bee pollen. Experiments were conducted with 3 independent variables and the responses comprised of antioxidant activity measured by the TPC, DPPH and FRAP assays.

Run	Independent Variables			Dependent Variables			
	Pulverisation	Solvent	Extraction	TPC	DPPH	FRAP	DPPH/FRAP Ratio
1	−	E70:30	A	20.86 ± 4.07	320.11 ± 27.00	342.28 ± 55.57	1.07
2	+	E70:30	A	20.85 ± 2.67	331.86 ± 43.20	357.36 ± 35.65	1.08
3	−	E70:30	M	19.99 ± 1.08	267.92 ± 39.77	396.39 ± 14.23	1.48
4	+	E70:30	M	19.42 ± 2.13	291.42 ± 56.06	349.19 ± 90.21	1.20
5	−	E70:30	R	21.37 ± 1.70	309.28 ± 32.62	326.54 ± 34.19	1.17
6	+	E70:30	R	20.46 ± 1.33	300.81 ± 48.42	328.85 ± 40.44	1.16
7	−	E70:30	S	18.80 ± 2.20	266.37 ± 56.46	326.61 ± 62.16	1.23
8	+	E70:30	S	19.68 ± 2.87	296.61 ± 55.53	338.49 ± 59.04	1.14
9	−	EtOH	A	16.61 ± 2.15	298.92 ± 14.55	286.19 ± 29.22	0.88
10	+	EtOH	A	16.92 ± 3.23	297.37 ± 15.99	289.85 ± 45.37	0.97
11	−	EtOH	M	17.03 ± 2.24	244.52 ± 55.49	302.95 ± 33.92	1.24
12	+	EtOH	M	16.84 ± 1.72	236.25 ± 52.65	297.27 ± 33.84	1.28
13	−	EtOH	R	19.35 ± 1.08	285.28 ± 32.63	323.98 ± 29.19	1.36
14	+	EtOH	R	18.84 ± 2.00	262.40 ± 37.24	310.51 ± 32.89	1.25
15	−	EtOH	S	15.67 ± 2.24	231.12 ± 52.88	269.77 ± 24.22	1.17
16	+	EtOH	S	16.57 ± 2.16	251.47 ± 37.37	286.61 ± 31.22	1.14
17	−	H_2_O	A	3.50 ± 0.52	34.67 ± 10.93	45.06 ± 12.36	1.30
18	+	H_2_O	A	3.68 ± 0.18	34.22 ± 6.14	44.05 ± 12.66	1.29
19	−	H_2_O	M	3.58 ± 0.55	20.91 ± 3.80	44.42 ± 6.33	2.13
20	+	H_2_O	M	3.34 ± 0.30	26.04 ± 10.26	43.01 ± 8.44	1.65
21	−	H_2_O	R	5.07 ± 1.45	49.21 ± 13.94	83.66 ± 23.00	2.83
22	+	H_2_O	R	4.98 ± 1.69	49.9 ± 20.24	80.89 ± 27.60	2.14
23	−	H_2_O	S	4.26 ± 0.29	31.72 ± 10.94	55.64 ± 9.42	1.75
24	+	H_2_O	S	4.35 ± 0.49	34.55 ± 4.53	56.99 ± 6.81	1.65
25	−	MtOH	A	19.55 ± 2.22	311.92 ± 33.70	196.83 ± 43.71	0.63
26	+	MtOH	A	19.25 ± 3.26	298.15 ± 47.53	237.14 ± 18.04	0.80
27	−	MtOH	M	19.17 ± 3.29	267.20 ± 62.25	221.76 ± 66.73	0.83
28	+	MtOH	M	18.58 ± 2.90	236.85 ± 46.00	268.84 ± 38.98	1.14
29	−	MtOH	R	21.15 ± 3.20	253.10 ± 25.66	254.02 ± 30.52	1.06
30	+	MtOH	R	20.00 ± 3.41	261.09 ± 41.84	270.53 ± 14.49	1.11
31	−	MtOH	S	17.85 ± 2.21	254.80 ± 65.14	216.45 ± 26.87	0.85
32	+	MtOH	S	17.95 ± 2.21	261.78 ± 70.17	241.74 ± 19.54	0.92

**Table 4:** Summary of regression coefficients. A[*n*] = pulverisation; B[*n*] = solvent type; C[*n*] = extraction process; AB[*n*] = interaction of pulverisation and solvent, B[*n*]C[*n*] = interaction of solvent type and extraction process. * = significant *p*-value < 0.05.

Coefficient Estimate			
Term	TPC	DPPH	FRAP
Intercept	3.76	13.71	14.64
A	-	-	0.1503
B[1]	0.7323 *	3.36 *	3.93 *
B[2]	0.3897 *	2.26 *	2.47 *
B[3]	−1.74 *	−8.15 *	−7.18 *
C[1]	−0.0195 *	0.9483 *	−0.4800 *
C[2]	−0.0691 *	−0.4900 *	0.0891 *
C[3]	0.1706 *	−0.2780 *	0.6496 *
AB[1]	-	-	−0.2061
AB[2]	-	-	−0.0267
AB[3]	-	-	−0.1826
B[1]C[1]	0.0937 *	-	0.6036 *
B[2]C[1]	−0.0348 *	-	−0.0579 *
B[3]C[1]	−0.1043 *	-	−0.3087 *
B[1]C[2]	0.0177 *	-	0.6213 *
B[2]C[2]	0.0365 *	-	0.1917 *
B[3]C[2]	−0.0886 *	-	−0.9382 *
B[1]C[3]	−0.0886 *	-	−1.14 *
B[2]C[3]	0.0498 *	-	0.0415 *
B[3]C[3]	0.0529 *	-	0.9468 *

## Data Availability

The data presented in this study are available as supplementary material.

## Supplementary Materials

The following are available online at https://www.mdpi.com/article/10.3390/antiox10071113/s1. Table S1: Data of pollen multilevel factor pollen analysis.
